# Supplementary material for: Genetic variation regulates opioid-induced respiratory depression in mice
Source: Sci Rep. 2020 Sep 11;10:14970. doi: 10.1038/s41598-020-71804-2 (PMC7486296; doi:10.1038/s41598-020-71804-2)

Genetic Variation Regulates Opioid-Induced Respiratory Depression in Mice

Jason A. Bubier[1*], Hao He[1], Vivek M. Philip[1], Tyler Roy[1], Christian Monroy Hernandez[1], Rebecca Bernat[2], Kevin D. Donohue[2,3] Bruce F. O'Hara[2,4], Elissa J. Chesler[1*]

[1]The Jackson Laboratory, Bar Harbor ME 04605;

[2]Signal Solutions, LLC, Lexington, KY;

[3]Electrical and Computer Engineering Department, University of Kentucky, Lexington, KY;

[4]Department of Biology, University of Kentucky, Lexington KY

*Corresponding author jason.bubier@jax.org

RUNNING TITLE:

KEY WORDS:

Figure S1

A

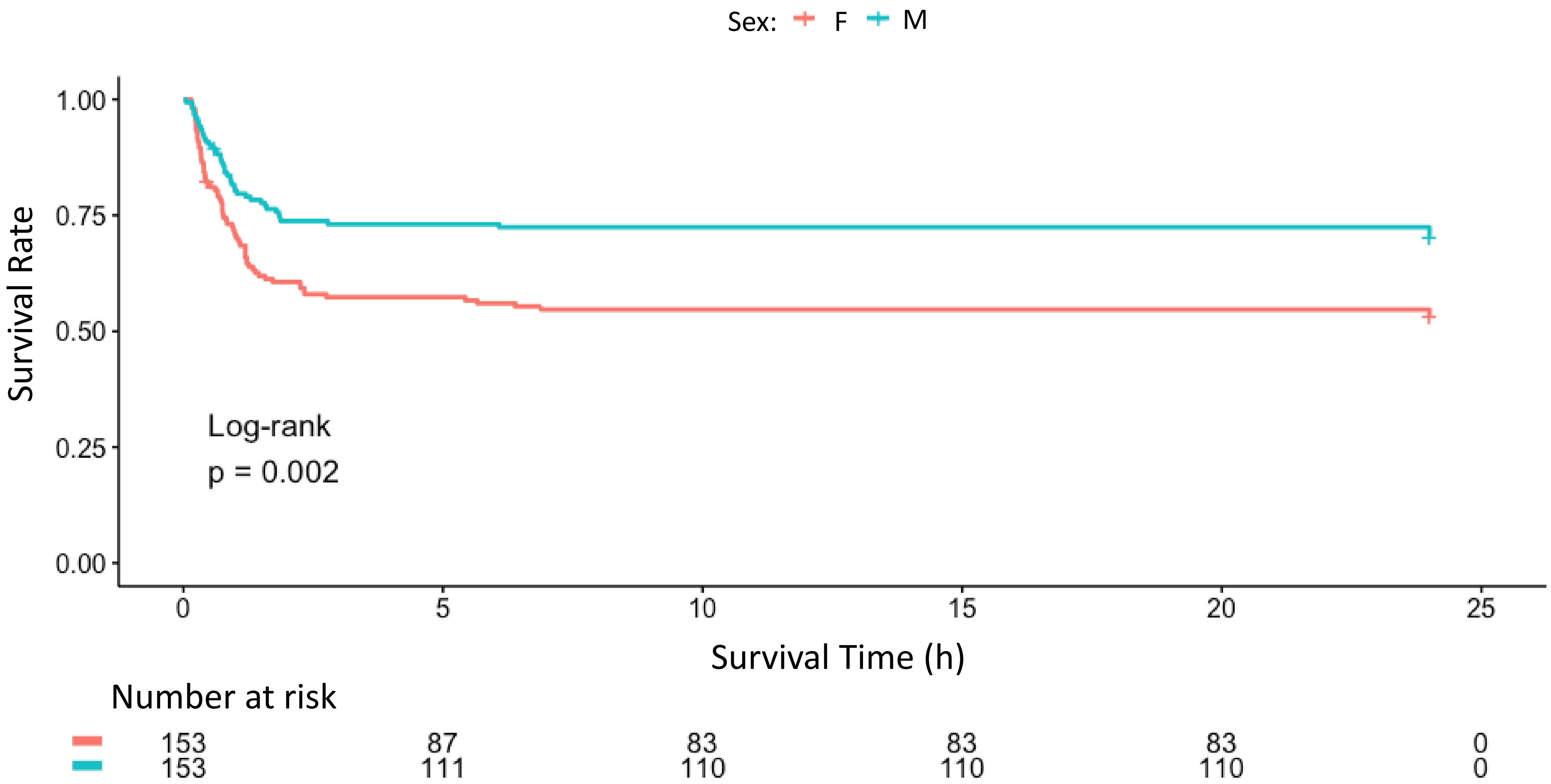
Sex: F M
1.00
0.75
0.50
0.25
0.00
Survival Rate
Log-rank
p = 0.002
0
5
10
15
20
25
Survival Time (h)
Number at risk
153 87 83 83 83 0
153 111 110 110 110 0

B

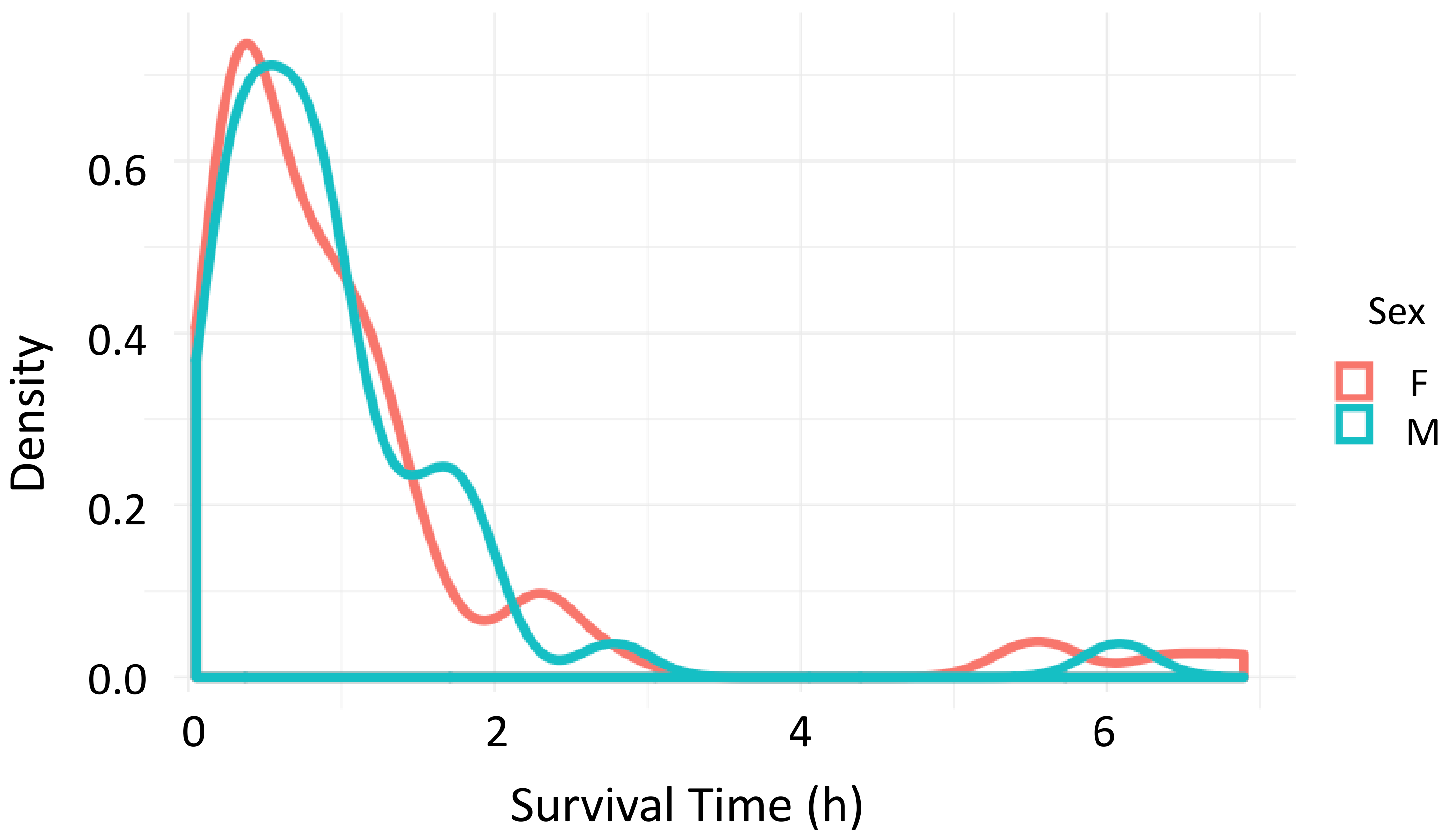
0.6
0.4
0.2
0.0
Density
0
2
4
6
Survival Time (h)
Sex
F
M

Supplemental Figure 1 C and D

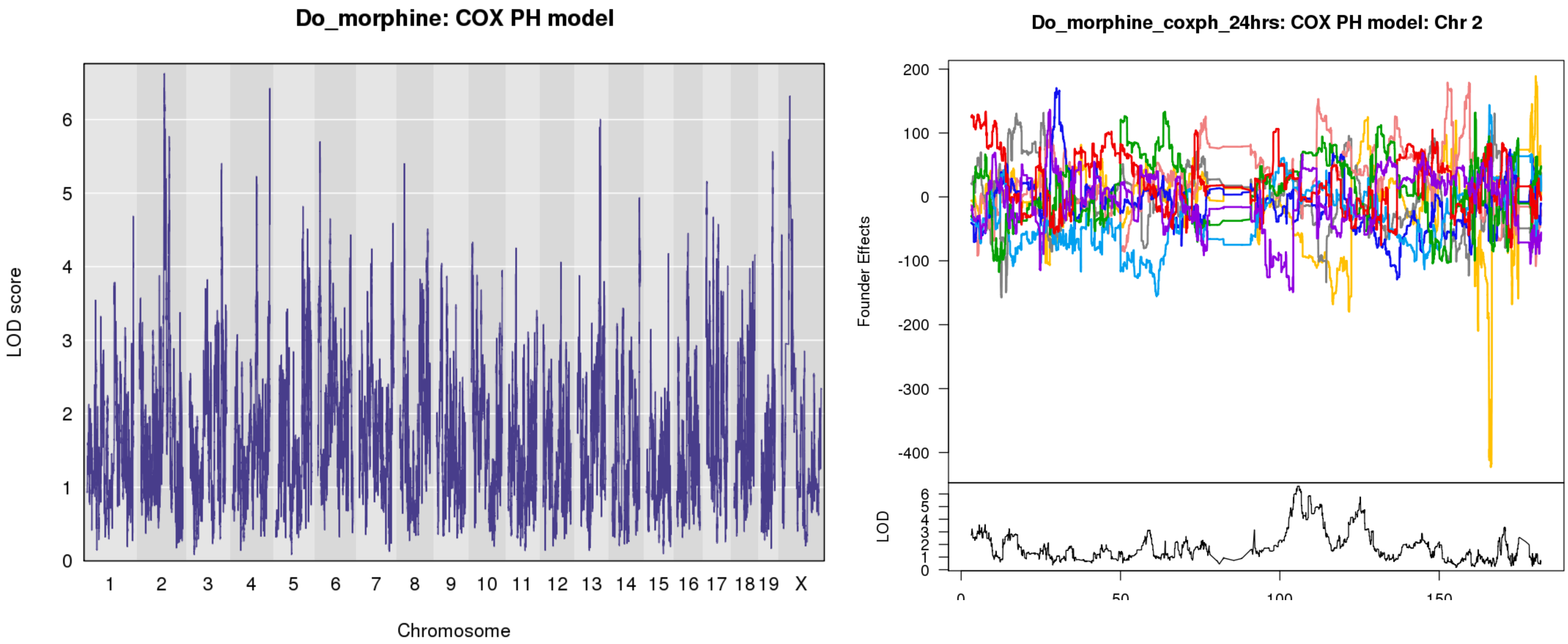

Supplement: Supplementary file 1 — Supplementary Figure 1 [file 41598_2020_71804_MOESM1_ESM.pdf]
