# Supplementary material for: Genetic variation regulates opioid-induced respiratory depression in mice
Source: Sci Rep. 2020 Sep 11;10:14970. doi: 10.1038/s41598-020-71804-2 (PMC7486296; doi:10.1038/s41598-020-71804-2)
Supplement: Supplementary file 4 — Supplementary legends [file 41598_2020_71804_MOESM4_ESM.docx]

**Figure S1. Mapping of the DO survival data using a Cox Proportional-Hazards Model** (**A**) Survival time of male (M, blue) and female (F, red) DO mice as represented by a Cox Proportional-Hazards Model. (**B**) The difference of these log likelihoods was taken and then divided by ln (10) to convert the result to the LOD scale. (**B**) Cox Proportional Hazards (COXPH) QTL mapping model which includes the genotype probabilities. (**D**) Allele effect plot of suggestive chromosome 2 locus. R/qtl2 https://kbroman.org/qtl2/

**Table S1**. The 1,885 SNPs that differ between NOD and WSB within the Chromosome 5 QTL interval.

**Table S2**. The 287 genes that are known targets for GALNT11 and expressed in the pre-Botzinger complex
